# Supplementary material for: Genome-wide identification and expression analysis of the trehalose-6-phosphate synthase (TPS) gene family in cucumber (Cucumis sativus L.)
Source: PeerJ. 2021 Apr 30;9:e11398. doi: 10.7717/peerj.11398 (PMC8092105; doi:10.7717/peerj.11398)
Supplement: Supplemental Information 1 [file peerj-09-11398-s001.docx]

Supplementary file 1. *TPS* genes were used to construct evolutionary tree.

| Species | Gene names | Gene ID | Database |
| --- | --- | --- | --- |
| *Arabidopsis thaliana* | *AtTPS1* | *At1g78580* | TAIR |
|  | *AtTPS2* | *At1g16980* |  |
|  | *AtTPS3* | *At1g17000* |  |
|  | *AtTPS4* | *At4g27550* |  |
|  | *AtTPS5* | *At4g17770* |  |
|  | *AtTPS6* | *At1g68020* |  |
|  | *AtTPS7* | *At1g06410* |  |
| *Glycine max*  *Oryza sativa*  *Cucumis sativus* | *AtTPS8*  *AtTPS9*  *AtTPS10*  *AtTPS11*  *GmTPS1*  *GmTPS2*  *GmTPS3*  *GmTPS4*  *GmTPS5*  *GmTPS6*  *GmTPS7*  *GmTPS8*  *GmTPS9*  *GmTPS10*  *GmTPS11*  *GmTPS12*  *GmTPS13*  *GmTPS14*  *GmTPS15*  *GmTPS16*  *GmTPS17*  *GmTPS18*  *GmTPS19*  *GmTPS20*  *OsTPS1*  *OsTPS2*  *OsTPS3*  *OsTPS4*  *OsTPS5*  *OsTPS6*  *OsTPS7*  *OsTPS8*  *OsTPS9*  *OsTPS10*  *OsTPS11*  *CsTPS1*  *CsTPS2*  *CsTPS3*  *CsTPS4*  *CsTPS5*  *CsTPS6*  *CsTPS7* | *At1g70290* *At1g23870*  *At1g60140*  *At2g18700* *Glyma15g2748*  *Glyma12g36280*  *Glyma08g12760*  *Glyma05g29651*  *Glyma20g25540*  *Glyma07g26980*  *Glyma12g15500*  *Glyma01g03870*  *Glyma02g03820*  *Glyma18g18590*  *Glyma17g07530*  *Glyma02g09480*  *Glyma04g35190*  *Glyma05g02020*  *Glyma06g19590*  *Glyma06g42820*  *Glyma08g39870*  *Glyma10g41680*  *Glyma13g01420*  *Glyma13g33970*  *LOC_Os05g44210*  *LOC_Os01g54560*  *LOC_Os01g53000*  *LOC_Os03g12360*  *LOC_Os02g54820*  *LOC_Os05g44100*  *LOC_Os08g31980*  *LOC_Os08g34580*  *LOC_Os09g25890*  *LOC_Os09g23350*  *LOC_Os09g20990*  *Csa_1G005560*  *Csa_1G467060*  *Csa_3G009420*  *Csa_4G622880*  *Csa_5G602180*  *Csa_6G520240*  *Csa_7G049190* | Phytozome  Rice Genome Annotation Project  EnsemblPlants |
