# Supplementary material for: Genome-wide identification and expression analysis of the trehalose-6-phosphate synthase (TPS) gene family in cucumber (Cucumis sativus L.)
Source: PeerJ. 2021 Apr 30;9:e11398. doi: 10.7717/peerj.11398 (PMC8092105; doi:10.7717/peerj.11398)
Supplement: Supplemental Information 2 [file peerj-09-11398-s002.docx]

| ID | Alignment start-end | Envelope start-end | hmm acc | hmm name | E-value |
| --- | --- | --- | --- | --- | --- |
| CsTPS1 | 54-539 | 53-540 | PF00982.21 | Glyco_transf_20 | 3.2e-181 |
|  | 589-823 | 589-824 | PF02358.16 | Trehalose_PPase | 1.0e-75 |
| CsTPS2 | 34-491 | 21-492 | PF00982.21 | Glyco_transf_20 | 2.7e-185 |
|  | 549-751 | 537-765 | PF02358.16 | Trehalose_PPase | 2.4e-55 |
| CsTPS3 | 63-546 | 62-548 | PF00982.21 | Glyco_transf_20 | 5.6e-185 |
|  | 597-829 | 597-830 | PF02358.16 | Trehalose_PPase | 1.7e-77 |
|  | 724-797 | 669-803 | PF08282.12 | Hydrolase_3 | 1.2e-05 |
| CsTPS4 | 95-560 | 94-561 | PF00982.21 | Glyco_transf_20 | 3.7e-188 |
|  | 618-814 | 606-834 | PF02358.16 | Trehalose_PPase | 5.2e-54 |
| CsTPS5 | 59-544 | 58-545 | PF00982.21 | Glyco_transf_20 | 9.8e-186 |
|  | 594-829 | 594-829 | PF02358.16 | Trehalose_PPase | 6.1e-78 |
|  | 723-793 | 695-799 | PF08282.12 | Hydrolase_3 | 2.8e-08 |
| CsTPS6 | 60-543 | 59-544 | PF00982.21 | Glyco_transf_20 | 1.4e-180 |
|  | 593-827 | 593-828 | PF02358.16 | Trehalose_PPase | 7.6e-75 |
|  | 721-802 | 714-819 | PF08282.12 | Hydrolase_3 | 1.7e-05 |
| CsTPS7 | 175-658 | 174-659 | PF00982.21 | Glyco_transf_20 | 3.8e-180 |
|  | 708-942 | 708-943 | PF02358.16 | Trehalose_PPase | 2.7e-73 |

Supplementary file 2. Domains in seven CsTPSs.
